# Impact of a Multidisciplinary Sepsis Initiative on Knowledge and Behavior in a Pediatric Center

**DOI:** 10.1097/pq9.0000000000000267

**Published:** 2020-03-10

**Authors:** Ryan K. Breuer, Amanda B. Hassinger

**Affiliations:** 1 From the Division of Pediatric Critical Care Medicine, Department of Pediatrics, John R. Oishei Children’s Hospital, Buffalo, N.Y.

## Abstract

**Objective:**

Our institution performed an educational initiative targeting previously identified barriers to pediatric sepsis recognition and treatment. We hypothesized that provider knowledge, attitude, and behavior would be improved 1 year after implementation.

**Methods:**

This was a prospective, observational study of a multi-faceted quality initiative introduced to providers in the Emergency Department, inpatient wards, and Pediatric Intensive Care Unit of a tertiary care children’s hospital. Educational platforms consisted of quarterly electronic sepsis “newsletters,” brightly colored posters highlighting protocol and screening strategies displayed throughout the hospital, and low-fidelity simulation sessions (drills) led by trained staff and incorporated into daily workflows. The content was driven by feedback from a baseline needs assessment of sepsis education. One year after implementation, the needs assessment was repeated.

**Results:**

Over 3 months, facilitators conducted 197 drills and captured a majority of nurses (89%), pediatrics residents (96%), and respiratory therapists (62%). By 6 months, 241 sessions had been completed. Approximately 55.4% of the 442 eligible staff participated in our post-intervention survey. Overall, knowledge of diagnostic criteria for pediatric sepsis and septic shock increased from pre-intervention levels (*P* = 0.015). Among post-implementation respondents, drill participants outperformed their colleagues (*P* = 0.001). A greater percentage of post-intervention respondents indicated comfort with sepsis recognition (*P* < 0.001), and fewer reported hesitating to bring sepsis concerns to their care team (*P* < 0.01).

**Conclusions:**

Our findings suggest that a multidisciplinary curriculum balancing active education—through brief, targeted simulation—and general awareness—through electronic resources and a poster campaign—can improve sepsis-related knowledge, attitude, and behavior among pediatric practitioners.

## Abstract 2

Supplemental Digital Content is available in the text.

## INTRODUCTION

The association between timely therapy and positive outcomes in pediatric sepsis has been reported for decades.^1–3^ Children with sepsis, who progress to critical illness and multiorgan dysfunction, are at risk for significant morbidity and mortality, often despite aggressive Pediatric Intensive Care Unit (PICU) intervention.^4^ While much is still unknown about the intricacies of sepsis in children, some degree of clinical decompensation may yet be preventable through improvement in diagnostic and therapeutic timeliness.^5,6^ Autopsy studies show that, despite a decline in overall diagnostic error rates, contemporary US hospitals still commit diagnostic errors in as many as 24.4% of patients, with up to 7% of such errors likely impacting outcome.^7^ Due to subtle presentations, septic children are at heightened risk for diagnostic delays. Of note, delays in diagnosis are still among the most commonly cited reasons for delays in therapy in health care.^8^

In response to several root cause analyses and quality assurance debriefings on index cases of sepsis, our institution sanctioned a hospital-wide educational needs assessment.^9^ Presuming our hospital was not immune to diagnostic and therapeutic delays, we sought to identify contributing factors that were potentially modifiable. The assessment focused on practitioner knowledge (ie, familiarity with published diagnostic criteria for pediatric sepsis), attitudes (assessed via self-reported comfort with bringing concerns to the care team), and behaviors (assessed via the self-reported frequency with which providers hesitated to do so).

Interestingly, all manner of providers (including faculty physicians) demonstrated at least some knowledge deficit, discomfort, and hesitation. These findings reflected those of smaller studies evaluating individual practitioner groups, which were at least partially amenable to targeted education.^10,11^ Such sepsis educational efforts have also been credited with reducing ICU admissions, lengths of stay, and mortality rates.^12,13^ These successes suggested that similar benefits might be derived from coordinated education on an institutional level.

Data from this initial assessment informed the design of a multidisciplinary educational initiative. Hospital leadership was supportive of this project as it aligned with their goal (and expectation) of institution-wide improvement in sepsis care. The objective of this study was to describe the impact of the educational initiative on hospital sepsis “culture,” examined through the lens of provider knowledge, attitude, and (self-reported) behavior, 1 year after implementation. We hypothesized that diagnostic knowledge and self-reported comfort among providers would be increased, while the frequency with which practitioners hesitated to act on suspicions of sepsis would be decreased.

## METHODS

This project was a prospective, observational study of the impact of an educational initiative on pediatric sepsis knowledge, attitudes, and behaviors in a freestanding children’s hospital. John R. Oishei Children’s Hospital (OCH, formerly the Women and Children’s Hospital of Buffalo) is a 200-bed teaching hospital serving Buffalo, N.Y., and its surrounding communities. It offers general and subspecialty services (apart from cardiothoracic surgery) to patients <21 years of age. Housestaff physicians are from both Categorical Pediatrics and Internal Medicine/Pediatrics. OCH admits ~12,000 children each year, of which roughly 1% are diagnosed with severe sepsis or septic shock.

### Impetus for the Initiative

In 2014, OCH’s Pediatric Quality Assurance (QA) Committee identified diagnostic and therapeutic delays in several cases of inpatient sepsis progressing to fulminant septic shock. It also identified an opportunity to improve staff awareness of the OCH Severe Sepsis Management Protocol, which had been put into practice 1 year earlier. Several months later, New York State mandated the reporting of hospital sepsis data. These events prompted 2 systemic changes: the creation of a separate Sepsis QA Committee and implementation of a cloud-based alert system operating through the hospital’s electronic health record (EHR) (St John Sepsis: Cerner Corporation, Kansas City, Mo.). Nurses would now be automatically notified if their patient’s data met pre-established criteria for Systemic Inflammatory Response Syndrome or sepsis. This system was limited by its triggers, which were based on adult sepsis research. Of note, no structured sepsis education accompanied these changes.

During this same period, PICU physician and nurse champions conducted a series of focus groups to determine staff perceptions of challenges to treating sepsis in children. Feedback from physicians, nurses (RNs), respiratory therapists (RTs), and advanced practice providers (APPs) helped inform the development of a needs assessment by these champions to describe practitioner knowledge, attitude, and behavior better. Knowledge was assessed through the creation of 7 clinical vignettes by the principal investigators (PIs). Each vignette provided a case stem, including examination findings and pertinent laboratory values, and prompted respondents to indicate if patients met the criteria for sepsis (or not) or septic shock (or not). Criteria published by Goldstein et al^14^ served as the standard for accuracy (**see Supplemental Digital Content 1** at http://links.lww.com/PQ9/A166). To aid with content validity, the PIs piloted the vignettes with 10 pediatric providers (3 faculty physicians, 3 RNs, 2 RTs, and 2 APPs) with at least 5 years of inpatient experience in an outside tertiary care enter. Likert items were provided for participants to indicate their level of comfort with recognizing sepsis and septic shock and the frequency with which they hesitate to bring sepsis/shock concerns to their care teams.

### Description of the Initiative

Survey responses indicated that all manner of providers in the Emergency Department (ED), wards, and PICU would benefit from sepsis education. No role appeared to be immune to deficits in diagnostic knowledge or to discomfort and hesitation when treating pediatric sepsis and septic shock. Participants did express a near-unanimous desire to improve, especially with the familiarity of diagnostic criteria and the strategies for early recognition and mobilization of resources for a timely therapy. PICU champions partnered with representatives from the ED and inpatient wards to produce a multi-tiered approach targeting physicians, RNs, RTs, and APPs from all 3 areas.

In January of 2016, the PIs gave a Grand Rounds presentation for the Department of Pediatrics, highlighting findings of the assessment and introducing the 3-tiered education plan. The first tier was electronic, with the creation of a “Severe Sepsis” order set in the EHR and a “Stop Septic Shock” portal on the OCH intranet. The order set pre-selected antibiotics, intravenous fluids, laboratory tests, and supportive care options aligning with the OCH Severe Sepsis Management Protocol. The webpage allowed users to access management protocols and their supporting literature and institutional sepsis epidemiologic data. It also would archive the newly minted sepsis e-newsletters, prepared by the PIs and emailed to staff quarterly. These contained Sepsis QA committee updates, tips and strategies for management, and quizzes for staff to use to assess their understanding of the material. The second tier was dedicated to visual marketing, with colored posters depicting sepsis screening and recognition tips and strategies placed throughout the hospital. Smaller versions were printed on identification badge-sized cards and distributed to staff. Laminated copies of the OCH Severe Sepsis Management Protocol were also placed at each nursing station and code cart. The third tier focused on active education through sepsis drills: 15-minute, low-fidelity simulation sessions facilitated by physician, nursing, and respiratory care champions. The target audience was ED, PICU and ward residents, RNs and RTs—those providers spending the most time at the bedside. These sessions were unscheduled, with the only stated goal of at least 1 drill per unit per month. Facilitators could engage their audience at a bedside or nursing station whenever they deemed appropriate, without the need for a manikin or other operating equipment. Instead, they presented a laminated card containing 1 of 15 clinical scenarios developed by the PIs (**see Supplemental Digital Content 2** at http://links.lww.com/PQ9/A167). Attendees were prompted to diagnose and articulate a management strategy, after which facilitators offered several preordained take-home points. Staff attendance was recorded. Multiple facilitators were assigned to each unit to capture both day and night practitioners.

### Follow-up Survey Distribution

In January 2017, 1 year after initiative implementation, a needs assessment was redistributed electronically to ED, PICU, and ward providers (**see Supplemental Digital Content 3** at http://links.lww.com/PQ9/A168). As with the initial survey, we excluded providers from Obstetrics/Gynecology, Anesthesia, Surgery, and Neonatology. Eligible staff were emailed a link to the survey webpage and consented to participate by opening the survey. Participation was voluntary, anonymous, and non-incentivized.

### Follow-up Survey Content

Demographic data collected included provider role, primary unit, and years in practice. Those practicing for 10 or more years were considered “experienced.” Providers were again given 7 vignettes to identify sepsis and septic shock correctly. Four scenarios were new to the post-initiative survey, while 3—those missed most often on the pre-intervention study—were repeated with slight modifications to the case presentations. Likert items were again provided to allow respondents to indicate their level of comfort with relaying suspicions of sepsis or shock to their care teams. Only respondents indicating strong agreement were considered “comfortable.” As in the original survey, respondents could also note the frequency with which they hesitated to do so for 4 specific reasons (eg, a desire to avoid “making a big deal in case they were wrong”). These reasons for hesitation were based on prominent, recurring themes from the focus groups. Participants indicating any frequency of hesitation, for any reason, were considered “hesitators.”

All surveys partially or entirely completed upon submission were accounted for when calculating the response rate.

### Statistical Analysis

We analyzed demographic data for normality using descriptive statistics; responses were described using proportions of all respondents, not solely those who answered each question (except the comfort and hesitation domains, so as not to potentially bias results by presuming negative attitudes or behaviors given there was no “right answer”). Respondents were stratified by role, reporting unit, and/or years of experience. Missing answers to scenario questions were considered incorrect. Chi-square or Fischer’s Exact tests compared proportions and *t* tests, or Mann-Whitney *U* tests compared continuous data between groups as determined by distribution. Regressions adjusted odds ratios for potential confounders, such as the impact of vignette performance, drill participation, experience, role, and comfort on reported behaviors. All statistics were performed using SPSS (IBM, Chicago, Ill., version 25.0) with significance set at a *P* value of 0.05.

The Institutional Review Board of the University at Buffalo approved this study.

## RESULTS

After 6 months of sepsis drills and a total of 12 months of a sepsis awareness campaign, we distributed a repeat, electronic survey to 442 OCH providers. A majority did participate, though the response rate (55.4%) was lower than that of the 2015 assessment (73.5%). Table 1 displays a breakdown of respondents to both surveys by role and experience. Most faculty (77%), residents (76%), nurses (56%), and RTs (81%) who participated in the post-intervention survey also took part in the initial assessment. Notably, all groups, except for RTs (contributing 8.6%, down from 16.2%, *P* = 0.008), contributed in similar proportions to both surveys.

**Table 1.**
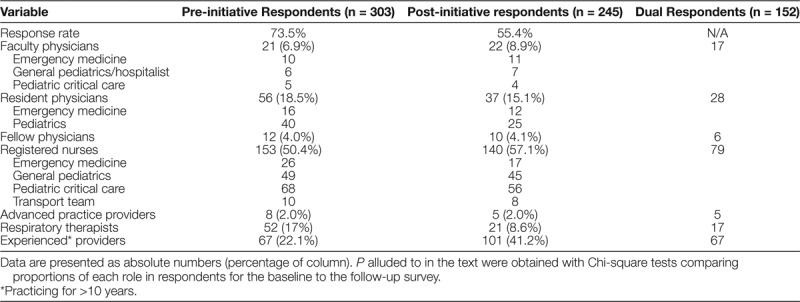
Breakdown of Respondents to Pre-, Post-initiative Assessments, as Well as Those Participating in Both of Provider Knowledge, Attitude, and Behaviors Related to Pediatric Sepsis

### Sepsis Drills

**Fig. 1.**
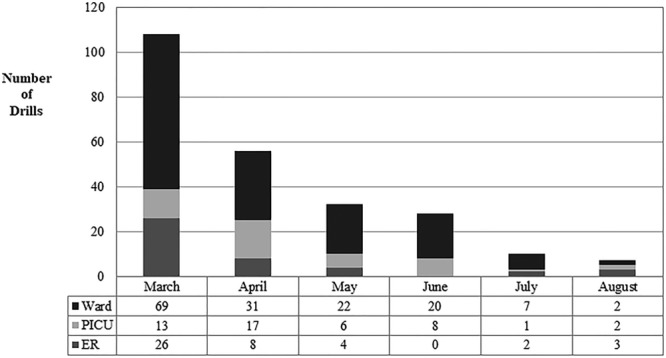
The number of sepsis drills performed by month and hospital unit.

**Fig. 2.**
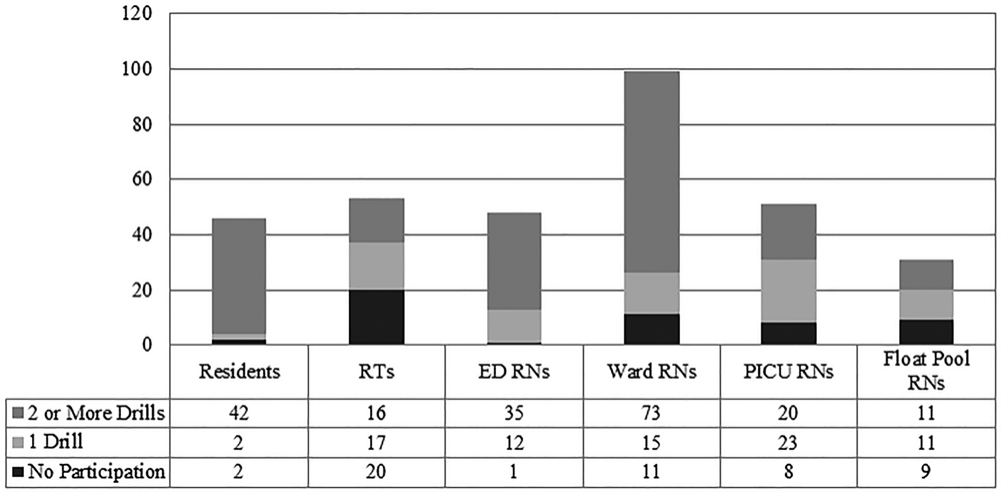
Overall sepsis drill participation by provider role.

A total of 46 physicians, RN and RT facilitators (28 for the inpatient wards, 10 for the ED, and 8 for the PICU) led 241 sepsis drills over 6 months. Figure 1 shows the relative contributions of each unit over time. Most drills (n = 196, 81.3%) were performed in the first 3 months, with the majority taking place on the inpatient wards. Figure 2 shows the overall participation totals by the provider role. Nearly all nurses from the ED (98%) and wards (89%), as well as the pediatric residents (96%), took part, with most completing more than 1 drill. While there was no plan to stop performing sepsis drills after 6 months, keeping our unit champions engaged and active (especially with other, non-sepsis initiatives implemented during this same timeframe) became practically difficult.

### Survey Findings—Comfort with Raising Sepsis Concerns

**Fig. 3.**
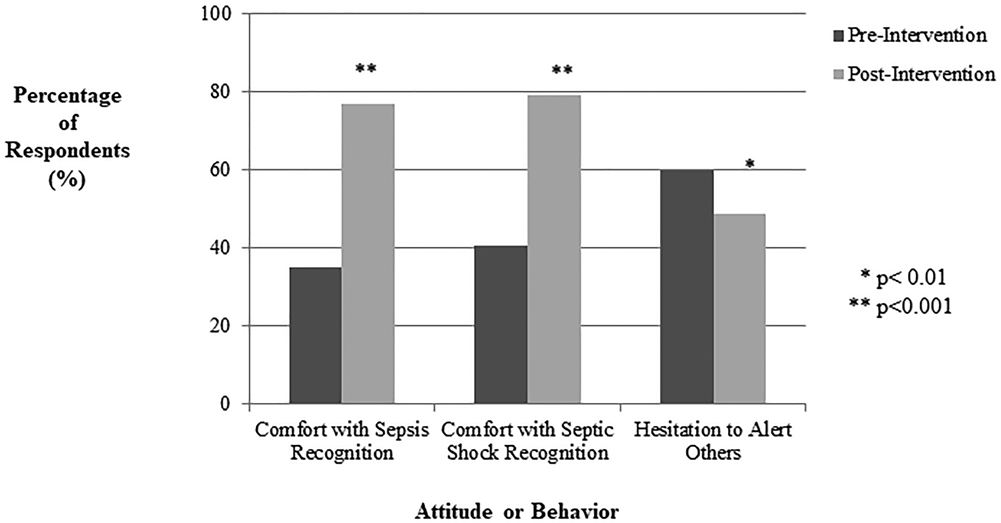
The proportion of survey respondents reporting comfort with bringing sepsis and septic shock concerns to their care team and indicating any hesitation to do so.

Figure 3 compares the percentage of pre- and post-intervention respondents who indicated they were comfortable (ie, strongly agreed with Likert items in this domain), bringing concerns about sepsis or septic shock to their care teams. Strong agreement increased significantly among post-intervention respondents for both entities (76.7% from 35.1% for sepsis, and 78.8% from 40.7% for septic shock; both *P* values < 0.001). Notably, those who participated in both surveys had higher odds of indicating strong agreement on the post-implementation assessment (OR 3.80, 95% CI, 2.50–5.88, *P* < 0.001).

### Survey Findings—Provider Behavior

Figure 3 also compares the percentage of pre- and post-intervention respondents reporting their hesitation to alert coworkers to concerns of possible sepsis or septic shock in a patient. The percentage of participants indicating any hesitation, for any of the 4 Likert items provided, decreased post-initiative (to 48.6% from 59.7%, *P* < 0.01). A lack of strong agreement in the comfort domain increased the odds that respondents would report hesitation in the behavior domain (OR 16.58, 95%CI, 7.72–35.62, *P* < 0.001).

New to the post-implementation survey, Likert items inquiring if respondents had ever refused to raise sepsis or shock concerns to their care team revealed that nearly 1 in 4 participants (24.7%) had done so at least once. We found no difference in refusal rate by provider role; however, inexperienced providers (in practice for fewer than 10 years) reported refusal more than their peers (43.9% versus 26.3%, *P* = 0.012). A majority of participants (62.5%) who indicated hesitation also indicated some frequency of refusal. Multivariate regression analyses found that resident physicians (aOR 4.91, *P* = 0.029) and those who did not report strong agreement in the comfort domain (ie, “uncomfortable” bringing sepsis concerns to others) had higher odds of reporting refusal (aOR 4.12, *P* = 0.014).

### Survey Findings—Diagnostic Knowledge

Respondents to the post-implementation survey, on average, answered a greater number of vignette questions correctly than respondents to the baseline needs assessment [4.03 (±2.4) versus 3.43 (±2.2), *P* = 0.015]. On average, participants in the follow-up survey who also took part in a sepsis drill answered more post-intervention questions correctly than those who did not [5.35 (±1.15) versus 4.67 (±1.41), *P* = 0.001]. However, even after excluding drill participants from the analysis, post-intervention respondents still outperformed pre-intervention respondents [4.67 (±1.41) versus 3.31 (±2.2), *P* < 0.001].

Within the post-implementation pool of respondents, those indicating strong agreement with comfort domain Likert items, on average, outperformed those who did not (4.28 versus 2.52, *P* < 0.001). A similar discrepancy was not found for those reporting clinical hesitation versus those who did not.

Three of the 7 vignettes—those missed most often in the pre-intervention survey—were rebranded in the post-intervention survey by modifying their case presentations. In 2 of the 3, a higher percentage of respondents correctly diagnosed the patients [29.1% to 39.3% (*P* = 0.043) and 51.2% to 65.9% (*P* = 0.005)]. For the third—a case of multiorgan failure but not cardiovascular dysfunction—a similar proportion of respondents rightly dismissed the diagnosis of septic shock (59.1% from 54.6%, *P* = 0.427).

## DISCUSSION

Provider comfort with pediatric sepsis and sepsis shock recognition and with bringing patient concerns to their care team, as well as the correct application of diagnostic criteria, all improved significantly following a hospital-wide education initiative. Also, fewer post-implementation respondents reported instances of actual hesitation to make their clinical concerns known. These findings suggest that a multidisciplinary campaign of small-group education and institutional awareness can positively impact sepsis knowledge, attitudes, and behavior among practitioners in a tertiary care center.

Small, low-fidelity, real-time simulation sessions led by physician and nurse facilitators were the centerpiece of this project’s provider education program. These drills emphasized sepsis content prioritized by the OCH respondents to our baseline needs assessment: reinforcement of diagnostic criteria, strategies for timely, bedside recognition, and identification of resources needed for prompt therapeutic intervention when indicated. Previous studies of individual provider groups (eg, resident physicians) have shown that structured educational modules—including those incorporating simulation—can increase sepsis knowledge and positively influence practitioner behavior.^15,16^ Simulation exercises and other means of “active participation” education have also been shown to improve coordination of care and affect practice change in pediatrics.^17–19^ However, the combination of this provider-centric strategy with a broader, institution-wide, multidisciplinary approach, to the best of our knowledge, has not been previously described. This multi-tiered methodology does make it difficult to ascertain what proportion of the improvements seen might be attributable to the sepsis drills as opposed to the poster campaign, EHR order set, screening cards, or e-newsletters. Post-intervention survey data (eg, the significant increase in vignette performance irrespective of drill participation) do suggest an impact of these other elements, especially considering the decrease in the number of drills performed after 3 months. Therefore, our results likely speak to the potential impact of a coordinated, multi-tiered strategy.

Our findings suggest a complex relationship between provider knowledge, comfort with raising clinical concerns, and hesitation (or failure) to do so. As previously described, sepsis knowledge—as indicated by vignette performance—significantly increased post-initiative, as did the proportion of respondents indicating comfort with calling attention to evolving cases of sepsis or septic shock. There was also an association between knowledge and comfort, with “comfortable” respondents answering, on average, more vignette questions correctly. This observation may be explained by the fact that providers who are better able to identify sepsis or septic shock are more confident in their suspicions and thus more comfortable reporting them to the care team. Interestingly, while “more knowledge” appeared to be associated with more comfort in raising concerns, it was not associated with less (self-reported) hesitation to do so. The lack of a difference in vignette performance between “hesitators” and “non-hesitators” suggests this behavior is influenced by more than mere comprehension. Perhaps the concerns reflected in the Likert items—inspired by pre-implementation focus group feedback—such as worry over the clinical implications of labeling a patient as septic (eg, additional venipunctures for lab work) are substantial enough to override providers’ instincts. It is also possible that respondents with “more knowledge” are hesitating for reasons other than their peers. For example, they may be overanalyzing cases and reluctant to call attention because their suspicions do not technically meet all necessary criteria. Future study is warranted, especially given the strong association between hesitation and refusal to raise concerns identified in the post-intervention survey.

This study has several limitations. It is a single-center study that may impact the external validity of the results. The simulation sessions and both surveys excluded neonatal, obstetrical, and surgical providers, given the nuances of sepsis particular to these patient populations. These exclusion criteria may, however, limit the generalizability of the results. Our evaluation strategies may also have introduced certain confounders. Vignette performance may not be the best surrogate for knowledge, and gains made could be due to the intervention better “priming” post-implementation respondents to recognize question patterns. If so, this may or may not be practically relevant, insofar as providers were still better able to make a cognitive association (eg, between viral, as well as bacterial infections, and the development of septic shock physiology). Knowledge improvements may also have been due to better clarity of the questions, although this was unlikely given that case presentations kept the same format. A post-initiative influx of providers well-versed in sepsis and septic shock could theoretically have impacted the results. Assessments of comfort and hesitation via Likert items may not be optimal, and all behavioral evaluations were based on self-reporting. Finally, we implemented this initiative after we established a Severe Sepsis QA Committee; the New York State’s Department of Health began mandating hospital outcome reporting, and we instituted a cloud-based EHR alert system. All of these may have raised awareness and impacted OCH sepsis culture. However, all pre-initiative survey findings were obtained with these factors already in place.

Results from several pediatric and adult sepsis quality initiatives have demonstrated the influence of bundled therapy or structured education on care delivery systems, process improvement and provider adherence to management guidelines.^20–23^ Some, but not all, show an impact on patient morbidity and mortality. This inconsistency may be due, at least in part, to the relative inattention paid to the provider mindset and institutional culture. Our findings suggest that both knowledge and attitude can benefit from sepsis education and awareness. How these changes relate to practitioner actions and patient outcomes is a topic for future study.

## CONCLUSIONS

To the best of our knowledge, this is the first study to evaluate the impact of a multidisciplinary educational initiative on sepsis knowledge, attitude, and behaviors in a tertiary care, freestanding children’s hospital. The combination of an awareness campaign and small-group education through low-fidelity sepsis drills increased familiarity with diagnostic criteria and practitioner comfort while decreasing the frequency of clinical hesitation when suspicious of sepsis in patients. However, the feasibility of sustaining these gains—as well as the complexity of the relationship between knowledge, attitude, and behavior—warrants future study.

## DISCLOSURE

The authors have no financial interest to declare in relation to the content of this article.

## Supplementary Material


