# Supplementary material for: Impact of a Multidisciplinary Sepsis Initiative on Knowledge and Behavior in a Pediatric Center
Source: Pediatr Qual Saf. 2020 Mar 10;5(2):e267. doi: 10.1097/pq9.0000000000000267 (PMC7190264; doi:10.1097/pq9.0000000000000267)
Supplement: Supplementary file 1 [file pqs-5-e267-s001.docx]

Supplement 3. Consensus definitions for pediatric sepsis and septic shock^#^

*Sepsis*: Proven or suspected infection with two or more of the following (at least one **must be** a temperature or leukocyte abnormality):

1. Core temperature of > 38.5°C or < 36°C
2. Abnormal heart rate:
   - Greater than 2 SD above age-specific mean (not attributable to external stimuli, medication or pain)

**OR**

- - Less than 10^th^ percentile for age (children under 1 year of age, not attributable to external stimuli or medication)

1. Abnormal respiratory rate:
   - Greater than 2 SD above age-specific normal

**OR**

- - Acute need for mechanical ventilation

1. Abnormal leukocyte count:
   - Elevated or depressed below age-specific normal (not attributable to chemotherapy)

**AND/OR**

- - Greater than 10% immature neutrophils

*Septic Shock*: Sepsis with cardiovascular dysfunction (despite 40mL/kg isotonic intravenous fluid given in 1 hour), as evinced by:

1. Blood pressure < 5^th^ percentile for age

**OR**

1. Need for vasoactive infusion to maintain age-appropriate blood pressure*

**OR**

1. At least 2 of the following:
   1. Base deficit of 5.0mEq/L or greater, not otherwise explained
   2. Arterial lactate twice the upper limit of normal
   3. Urine output < 0.5mL/kg/h
   4. Capillary refill > 5 seconds
   5. Core-to-peripheral temperature gradient > 3°C

SD = Standard deviation

*Dopamine dose > 5mcg/kg/min, any dose of epinephrine, norepinephrine, dobutamine

|  | Abnormal Value | | | | |
| --- | --- | --- | --- | --- | --- |
| Age | Tachycardia  (beats/min) | Bradycardia  (beats/min) | Respiratory Rate  (breaths/min) | Leukocyte Count  (x 10^3^/mm) | Systolic Blood Pressure  (mm Hg) |
| Less than 1 week | > 180 | < 100 | > 50 | > 34 | < 65 |
| 1 week - 1 month | > 180 | < 90 | > 40 | > 19.5 or < 5 | < 75 |
| 1 month – 1 year | > 180 | < 90 | > 34 | > 17.5 or < 5 | < 100 |
| 2-5 years | > 140 | N/A | > 22 | > 15.5 or < 6 | < 94 |
| 6-12 years | > 130 | N/A | > 18 | > 13.5 or < 4.5 | < 105 |
| 13 to 18 years | > 110 | N/A | > 14 | > 11 or < 4.5 | < 117 |

Lower values for heart rate, leukocyte count, systolic blood pressure are 5^th^ percentile for age

Upper values for heart rate, respiratory rate, leukocyte count are 95^th^ percentile for age

^#^Goldstein B, Giroir B, Randolph A, and the Members of the International Consensus Conference on

Pediatric Sepsis. *Pediatr Crit Care Med*. 2005;6(1):2-8.
