# Supplementary material for: Impact of a Multidisciplinary Sepsis Initiative on Knowledge and Behavior in a Pediatric Center
Source: Pediatr Qual Saf. 2020 Mar 10;5(2):e267. doi: 10.1097/pq9.0000000000000267 (PMC7190264; doi:10.1097/pq9.0000000000000267)
Supplement: Supplementary file 2 [file pqs-5-e267-s002.pdf]

Scenario 1

You are called to the room of Gregory, a 12 year old boy with developmental delay and autism, by his mother. She is worried because he is "starting to look worse" than a few hours ago and hasn't urinated since admission.

History of Present Illness

Came to the ED with "trouble breathing" that got him sent home early from school.
Tactile fever last night, but felt/looked 'ok' this morning per Mom so got on the bus.
No home medications or allergies.

ED Course

No IV as PO intake reportedly 'close to normal' at home
Labs sent, given Tylenol for 38.9°C orally
Rapid screen for influenza A was *positive*
Needed O_2 by facemask intermittently so admitted to monitor respiratory status

Current Vital Signs

Temperature: 39.1°C (Axillary)   Heart Rate: 155   Blood Pressure: 122/94 SpO_2: 94% (Room Air)   Respiratory rate: 32

Physical Exam

Appears agitated, making groaning sounds that Mom says are a little worse than baseline
Breathing quickly, appears labored with use of accessory muscles
Pulses thready, 1+ in right/left wrists, capillary refill 6 seconds

Labs

WBC count of 19.2, 80% lymphocytes
Sodium 145, K 5.4, Cl 100, HCO3 16, BUN 20, SCR 0.8, glucose 80

What does this patient have? **SIRS (high HR and fever)** + viral infection = **SEPSIS**
SEPSIS + **cardiovascular dysfunction (poor UOP and delayed CR)** + **depressed mental status** = **SEVERE SEPSIS**

Interventions: Start the SEVERE SEPSIS PROTOCOL:

UB|MD PEDIATRICS

Women and Children's Hospital of Buffalo
Pediatric Severe Sepsis Protocol

Women & Children's Hospital of Buffalo
A Kaleida Health Facility

Minutes		Goals for time 0-15 min (check off the box as completed):	Performed by:
0 to 15	☐	Cardiac monitor attached	ALL
	☐	Supplemental oxygen started via facemask at 100%	ALL
	☐	Start sepsis order set	RN, RRT
	☐	Peripheral intravenous (PIV) line started and draw labs	RRT, MD, RN
	☐	Place Intraosseous (IO) line if no PIV after 3 attempts or **10 minutes**	MD
	☐	STAT labs: blood culture, CBC with differential, VBG, glucose, electrolytes, lactate, iStat	RN, RRT
	☐	Administer 1st 20cc/kg NS or LR IV/IO bolus via push/pull over 10 min	RN

STOP

Recheck vitals and perfusion

If still abnormal or patient ill appearing, continue protocol	If vitals normalized and patient well appearing, discontinue protocol.

⬇

Key Learning Points

1. "Sepsis" just means 'SIRS' with an infection. It does not matter if that infection is viral, bacterial, or fungal!

2. Children with developmental problems can be hard to diagnose with sepsis. Some signs and symptoms (high heart rate, agitation) might be 'normal for them' sometimes. This just means we need to keep an even closer eye on these patients.

3. Trust your instincts! Almost everyone gets tachycardia and/or tachypnea when they have a fever. **Ask about mental status, urine output, mottling, and perfusion.** These should NOT change much at all with a simple fever.

SYSTEMATIC INFLAMMATORY RESPONSE SYNDROME (SIRS)			SEPSIS	→ Organ Failure Begins →	SEVERE SEPSIS	SEPTIC SHOCK
↑ or ↓ WBC<br>OR<br>Bands >10%<br>OR<br>Temp <36, >38.5	+	Elevated HR<br>OR<br>Elevated RR	SIRS<br>+<br>ANY INFECTION		SEPSIS<br>+<br>Cardiovascular dysfunction<br>OR<br>Respiratory failure<br>OR<br>≥2 organ failures†	SEPSIS<br>+<br>Cardiovascular dysfunction*<br><br>*(*after adequate fluid resuscitation)*

Systemic Inflammatory Response Syndrome (SIRS) criteria:

Age group	HR	RR	WBC	SBP<5th %ile
**0-7 days**	>180 or <100	>50	>34	<60
**8-30 days**	>180 or <100	>40	<5 or >19.5	<65
**31 days to <2 years**	>180 or <90	>34	<5 or >17.5	<70
**2 to <6 years**	>140	>22	<6 or >15.5	<75
**6 to <13 years**	>130	>18	<4.5 or >13.5	<85
**>13 years**	>110	>14	<4.5 or >11	<90

Goldstein et al. CCM 2005;6(1):2-6.

Organ Failure Criteria†

This organ is affected if **any** of the following occur:

**Cardiovascular**	SBP < 5th %ile for age	**OR**<br>2 or more of the following:	Base Deficit > -5 mEq/L	Lactate > 4mmol/L	Urine <0.5ml/kg/hr	Capillary refill > 5 sec
**Respiratory**	Needing CPAP or BIPAP or invasive mechanical vent	**OR**<br>FiO2>50% to keep SpO2>92%	**OR**<br>PaCO2>65 mmHg OR PvCO2 > 70	**OR**<br>PaO2:FiO2 ratio ≤300		
**Kidney**	SCr > 2 times normal for age	**OR** SCr > 2 times higher than baseline				
**Liver**	Tbili > 4mg/dL	**OR** ALT > 2 times higher than normal for age				
**Hematologic**	Platelets < 80x 10^3/mcL	**OR** INR > 2				
**Neurologic**	GCS ≤ 11 without sedation	**OR** Decrease in mental status from baseline				

Age	Excessive tachycardia when at each body temperature								SBP 5%ILE FOR AGE
	**37.8**	**38.3**	**38.9**	**39.4**	**40**	**40.6**	**41.1**	**41.7**	
< 2 years *	>180	>185	>190	>195	>200	>205	>210	>215	*<30 days: 60*<br>*1mo- 2y: 70*
2 - 5 years	>140	145	150	155	160	165	170	175	75
6 - 12 years	>130	135	140	145	150	155	160	165	85
13 - 18 years	>110	115	120	125	130	135	140	145	90

*Also qualify if bradycardic:	< 1 month: HR <100	1 - 23 months: HR < 90
