# Supplementary material for: Impact of a Multidisciplinary Sepsis Initiative on Knowledge and Behavior in a Pediatric Center
Source: Pediatr Qual Saf. 2020 Mar 10;5(2):e267. doi: 10.1097/pq9.0000000000000267 (PMC7190264; doi:10.1097/pq9.0000000000000267)
Supplement: Supplementary file 3 [file pqs-5-e267-s003.pdf]

WCHOB Sepsis Survey

Please complete the survey below.

Thank you!

What is your role in patient care?

◯ Physician - Resident
◯ Physician - Fellow
◯ Physician - Attending
◯ Nurse
◯ Respiratory Therapist
◯ Nurse Practitioner
◯ Physician's Assistant

Did you take the original PRESS (sepsis) survey?

◯ Yes
◯ No

Attending Physicians Please indicate your division:

◯ Emergency Medicine ◯ General Pediatrics ◯ Critical Care

Resident PhysiciansPlease indicate your division:

◯ Emergency Medicine ◯ General Pediatrics

Fellow PhysiciansPlease indicate your division:

◯ Emergency Medicine ◯ Critical Care

Residents and FellowsWhat is your current level of training?

◯ PGY-1 ◯ PGY-2 ◯ PGY-3 ◯ PGY-4 ◯ PGY-5 ◯ PGY-6 ◯ PGY-7 or greater

---

Nurses What is your primary care team/area?*If you split time, choose where you spend the majority*

◯ Emergency Department ◯ Inpatient General Pediatrics wards ◯ PICU ◯ STAT team
◯ Other

Respiratory TherapistsWhat is your primary care team/area?*If you split time, choose where you spend the majority*

◯ Emergency Department ◯ Inpatient General Pediatrics wards ◯ PICU ◯ STAT team
◯ Other

Nurse Practitioners and Physician's AssistantsWhat is your primary care team/area?

◯ Emergency Department ◯ PICU ◯ Other

Other:

____

How many years have you been in practice (out of school/training)?

◯ Less than 3
◯ 3-6
◯ 7-9
◯ 10 or more

How many years have you been at Women and Children's Hospital of Buffalo (training and/or in practice)?	◯ Less than 3<br>◯ 3-6<br>◯ 7-9<br>◯ 10 or more
How do you think Women and Children's Hospital of Buffalo compares to other U.S. pediatric centers in sepsis care?	◯ Among the best (top 10%)<br>◯ Above average (upper 25%)<br>◯ Average<br>◯ Below average (lower 25%)<br>◯ Among the worst (bottom 10%)
How do you think Women and Children's Hospital of Buffalo compares to other U.S. pediatric centers in septic shock care?	◯ Among the best (top 10%)<br>◯ Above average (upper 25%)<br>◯ Average<br>◯ Below average (lower 25%)<br>◯ Among the worst (bottom 10%)
Does Women and Children's Hospital of Buffalo have a protocol for care of patients with severe sepsis or septic shock?	◯ Yes<br>◯ No<br>◯ Not sure
Does Women and Children's Hospital of Buffalo have a specialized order set for patients with severe sepsis or septic shock?	◯ Yes<br>◯ No<br>◯ Not sure
How often have you used the severe sepsis order set in the care of your patients?	◯ Never<br>◯ Rarely<br>◯ Sometimes<br>◯ Frequently<br>◯ Always
Which are some reasons you have not used the severe sepsis order set in the past?Select all that apply	☐ What's a sepsis order set?<br>☐ I forget<br>☐ Not easy to find the order set in the EMR<br>☐ Not easy to find what I need when I open the order set<br>☐ Already doing most/all of what it says already<br>☐ My patients, though technically "septic," are not sick enough to warrant all those labs/meds/interventions<br>☐ Other
Other:	__________________________________
Which of the following do you think make it difficult to recognize sepsis and/or septic shock in children?*Choose all that apply	☐ Viral infection<br>☐ Lack of a positive culture<br>☐ Developmental delay<br>☐ Complex medical history<br>☐ Many previous admissions for similar issues<br>☐ Kids have a wide range of "normal" vitals, labs<br>☐ Coworker discouragement<br>☐ Easy to dismiss signs/symptoms (e.g. fever 'not real' in a bundled neonate)<br>☐ Diagnostic fatigue ("Everything looks like sepsis")<br>☐ Lack of familiarity with the diagnostic criteria<br>☐ Other
Other:	__________________________________

Please indicate your level of agreement with the following statements:

	Strongly Disagree	Somewhat Disagree	Neither agree nor disagree	Somewhat Agree	Strongly Agree
Women and Children's Hospital of Buffalo excels at diagnosing sepsis	○	○	○	○	○
Women and Children's Hospital of Buffalo excels at diagnosing septic shock	○	○	○	○	○
Women and Children's Hospital of Buffalo excels at treating sepsis	○	○	○	○	○
Women and Children's Hospital of Buffalo excels at treating septic shock	○	○	○	○	○
Providers in Women and Children's Hospital of Buffalo use clear and consistent definitions of sepsis/septic shock	○	○	○	○	○

How often do you HESITATE TO NOTIFY other providers about a possible sepsis/septic shock patient for the following reasons...

	Never	Rarely	Sometimes	Frequently	Very frequently/Always
Concerns over a negative response from coworkers	○	○	○	○	○
Prior discouragement from making these diagnoses	○	○	○	○	○
Worries about alarming patients, families, or co-workers	○	○	○	○	○
Worries about making "too big a deal" in case you are mistaken	○	○	○	○	○
Wish to avoid using these "labels"	○	○	○	○	○

How often do you CHOOSE NOT TO NOTIFY other providers about a possible sepsis/septic shock patient for the following reasons...

	Never	Rarely	Sometimes	Frequently	Very Frequently/Always
Concern over a negative response from coworkers	○	○	○	○	○
Prior discouragement from making these diagnoses	○	○	○	○	○
Worry about alarming patients, families, or co-workers	○	○	○	○	○
Worry about making "too big a deal" in case you are mistaken	○	○	○	○	○
Wish to avoid using these "labels"	○	○	○	○	○

Please indicate your level of agreement with the following statements:
"I AM COMFORTABLE...

	Strongly Disagree	Somewhat Disagree	Neither Agree or Disagree	Somewhat Agree	Strongly Agree
...alerting a COWORKER my patient might be septic"	○	○	○	○	○
...alerting a SUPERVISOR or ATTENDING PHYSICIAN my patient might be septic"	○	○	○	○	○
...alerting a COWORKER my patient might be in septic shock"	○	○	○	○	○
...alerting a SUPERVISOR or ATTENDING PHYSICIAN my patient might be in septic shock"	○	○	○	○	○
...identifying septic shock in my patient when their blood pressure is NORMAL"	○	○	○	○	○
...identifying septic shock in my patient when their blood pressure is ABNORMAL"	○	○	○	○	○

You are called to the bedside of each of the following patients. Based on your understanding of the pediatric criteria, please indicate whether each has sepsis.

3 y/o male with nephrotic syndrome admitted for worsening edema, acute on chronic renal failure secondary to medication non-compliance.Temperature: 38.7°C orallyHeart rate: 150 beats/minRespiratory rate: 50 breaths/minBlood pressure: 140/95Capillary refill: 4-5 seconds, mottled with pitting edemaPulses: 2+ central, 1+ peripheralWBC count: 14.4, with 70% neutrophils, 20% lymphocytesUrinalysis: 3+ protein, 3+ hemoglobin, many WBC, + leuk esterase, + nitrites, many bacteria

◯ Yes, this patient has sepsis
◯ No, this patient does not meet sepsis criteria
◯ Unsure

---

6 year old male s/p MVA (motor vehicle accident), pedestrian vs. car. Admitted with liver laceration to the ICU for serial blood counts. No other injuries.Temperature: 38.1°C orallyHeart rate: 175 beats/minRespiratory rate: 36 breaths/minBlood pressure: 132/60Capillary refill: 3 secondsPulses: 3+, central and peripheralWBC count: 5.7, with 88% neutrophils, 10% lymphocytesHemoglobin: 6.3

◯ Yes, this patient has sepsis
◯ No, this patient does not meet sepsis criteria
◯ Unsure

---

14 year old male with influenza A on viral screening. Bacterial cultures negative, no antibiotics. Temperature: 39.0°C orallyHeart rate: 165 beats/minRespiratory rate: 18 breaths/minBlood pressure: 110/65Capillary refill: < 2 secondsPulses: 2+, central and peripheralWBC count: 7.4, with 70% lymphocytes, 20% neutrophils

◯ Yes, this patient has sepsis
◯ No, this patient does not meet sepsis criteria
◯ Unsure

---

12 y/o male with global developmental delay and autism, admitted for "trouble breathing" and hypoxemia. Improved on nasal cannula, now on 2L/min. Temperature: 39.1°C orallyHeart rate: 155 beats/minRespiratory rate: 32 breaths/min, SpO2: 95%Blood pressure: 122/94Capillary refill: 5 secondsPulses: 1+, central and peripheralWBC count: 19.2, with 80% lymphocytes, 16% neutrophils

◯ Yes, this patient has sepsis
◯ No, this patient does not meet sepsis criteria
◯ Unsure

You are called to the bedside to evaluate the following patients. If all have received 40ml/kg of intravenous fluids in the last hour, would they meet the criteria for SEPTIC SHOCK based on the information provided?

3 y/o male with pneumococcal pneumonia on high-flow nasal cannula. Appears in mild distress, minimal accessory muscle use and subcostal retractions. Tired and cranky but appropriately interactive.Temperature: 38.8°C rectally, 38.5°C axillaryHeart rate: 110 beats/minRespiratory rate: 20 breaths/minBlood pressure: 110/50Urine output of 1.5mL/kg/hr over last 12 hoursWBC count: 18.1, with 85% neutrophils, 12% immature neutrophils (bands)Arterial blood gas: pH of 7.39, CO2 of 40, base deficit of -0.5Platelets: 75,000/mm3Serum creatinine: 1.2 mg/dL (baseline 0.5 mg/dL)

◯ Yes, this patient has septic shock
◯ No, this patient does not meet criteria for septic shock
◯ Unsure

6 year old female with a history of ALL (acute lymphoblastic leukemia), receiving chemotherapy. Admitted with one day of fever and vomiting. Sleepy but answers questions appropriately.Temperature: 39.7°C orally, 36.5°C axillaryHeart rate: 120 beats/minRespiratory rate: 18 breaths/minBlood pressure: 108/60Capillary refill: 6 secondsUrine output of 0.4mL/kg/hr over last 12 hoursWBC count: 0.4Arterial blood gas: pH of 7.30, CO2 of 32, base deficit of -6Platelets of 100,000/mm3Serum creatinine normal

◯ Yes, this patient has septic shock
◯ No, this patient does not meet criteria for septic shock
◯ Unsure

19 day old female with RSV, on ampicillin and cefotaxime while blood, urine, and CSF cultures pending. Temperature: 38.7°C rectallyHeart rate: 190 beats/minRespiratory rate: 48 breaths/minBlood pressure: 90/44Capillary refill: > 5 secondsPulses: 1+, central and peripheral, mottled skinUrine output of 0.2mL/kg/hr over last 12 hoursWBC count: 32.6 with 82% neutrophils, 10% lymphocytesArterial blood gas: pH of 7.33, CO2 of 22, base deficit of -9Platelets of 280,000/mm3Serum creatinine 0.7

◯ Yes, this patient has septic shock
◯ No, this patient does not meet criteria for septic shock
◯ Unsure

Please answer the next few questions about sepsis drills and our efforts to date so we can better serve you and our patients:

Have you participated in any sepsis drills in the past year?	◯ Yes<br>◯ No
Approximately how many?	◯ 1 or 2<br>◯ 3-5<br>◯ 6 or more
How valuable do you feel sepsis drills are to our education?	◯ Very much<br>◯ Moderately<br>◯ Somewhat<br>◯ Little<br>◯ Not at all
What have you learned from your participation in sepsis drills?Choose all that apply	☐ Definitions for sepsis, severe sepsis and septic shock<br>☐ That we have a severe sepsis protocol at WCHOB<br>☐ What the severe sepsis protocol contains<br>☐ How to recognize early signs of severe sepsis or septic shock<br>☐ How to treat patients with severe sepsis or septic shock<br>☐ Nothing, I already knew everything discussed<br>☐ Other
Other:	__________________________________
Which of the following would be helpful educational platforms?Choose all that apply	☐ Grand Rounds<br>☐ Online modules/quizzes<br>☐ Talent Management<br>☐ Small-group discussions<br>☐ More sepsis drills!<br>☐ Simulation/Mock code sessions<br>☐ Other
Other:	__________________________________

In your experience, how have the following aspects of the sepsis "culture" at Women and Children's Hospital of Buffalo changed over the past year?

	Improved significantly	Improved somewhat	No change	Gotten somewhat worse	Gotten significantly worse
Sepsis and shock awareness	◯	◯	◯	◯	◯
Prompt sepsis recognition	◯	◯	◯	◯	◯
Prompt shock recognition	◯	◯	◯	◯	◯
Prompt sepsis treatment	◯	◯	◯	◯	◯
Prompt shock treatment	◯	◯	◯	◯	◯
Collaboration and communication about sepsis between providers	◯	◯	◯	◯	◯
Staff education	◯	◯	◯	◯	◯
Overall quality of care of patients with sepsis	◯	◯	◯	◯	◯
